# Human Epidermal Growth Factor Receptor 2 Overexpression and Amplification in Patients With Colorectal Cancer: A Large-Scale Retrospective Study in Chinese Population

**DOI:** 10.3389/fonc.2022.842787

**Published:** 2022-04-28

**Authors:** Shujuan Ni, Xin Wang, Jinjia Chang, Hui Sun, Weiwei Weng, Xu Wang, Cong Tan, Meng Zhang, Lei Wang, Zhaohui Huang, Dan Huang, Midie Xu, Weiqi Sheng

**Affiliations:** ^1^ Department of Pathology, Fudan University Shanghai Cancer Center, Shanghai, China; ^2^ Department of Medical Oncology, Shanghai Medical College, Fudan University, Shanghai, China; ^3^ Institute of Pathology, Fudan University, Shanghai, China; ^4^ Department of Medical Oncology, Fudan University Shanghai Cancer Center, Shanghai, China; ^5^ Wuxi Cancer Institute, Affiliated Hospital of Jiangnan University, Wuxi, China

**Keywords:** colorectal cancer, HER2, methodology, amplification, prognosis

## Abstract

**Background:**

Cumulative evidence in colorectal cancer (CRC) suggests that patients with human epidermal growth factor receptor 2 (HER2) overexpression or amplification can benefit from anti-HER2 therapy. The purpose of our study was to evaluate HER2 status and its correlation with clinicopathological characteristics and survival according to currently utilized HER2 diagnostic criteria in a large cohort of Chinese CRC patients.

**Methods:**

HER2 protein expression was tested by immunohistochemistry (IHC) in formalin-fixed, paraffin-embedded (FFPE) samples from 4,836 CRC patients in our institution. Breast cancer (BC) and gastroesophageal adenocarcinoma (GEA) criteria, as well as the HERACLES criteria, were used for the determination of HER2 status. Dual-color silver-enhanced *in situ* hybridization (DSISH) was performed in all IHC 2+~3+ cases determined by BC/GEA criteria.

**Results:**

The HER2 expression rate of IHC (1+~3+) was 7.01% (339/4,836) and 6.02% (291/4,836) in CRCs based on the BC/GEA criteria and the HERACLES criteria, respectively, while combined DSISH results in the HER2 amplification/overexpression ratio of 3.39% (164/4,836) in our cohort. HER2 expression detected by IHC was positively correlated with the female gender, whereas the HER2 overexpression/amplification showed no correlation with any clinicopathological parameter. In addition, no significant correlation was found between HER2 statuses and either disease-free survival or overall survival regardless of the evaluation criterion used. However, patients with HER2 1+ CRC showed a tendency of having the shortest overall survival as compared with any other group of patients according to the HERACLES criteria, and this trend has always existed in the rectal location, T3 stage, and TNM stage II, medium differentiation, and perineural invasion stratified group. Furthermore, the HER2 protein expression was significantly negatively correlated with RAS/BRAF mutations according to the HERACLES criteria.

**Conclusion:**

To our knowledge, this is the largest study of HER2 status in Asian patients with CRC. Our findings suggest that the current most commonly used HERACLES criteria might be too strict for patients with CRC. Future studies are needed to explore the most suitable criteria for screening CRC patients who could benefit from anti-HER2 therapy as much as possible.

## Introduction

Colorectal cancer (CRC) is the third most prevalent cancer and the second most common cause of cancer-related death in the world, with an annual death rate exceeding 608,700 according to the National Cancer Institute. According to the latest data released by the International Agency for Research on Cancer (IARC) of the WHO in 2020, CRC ranks as the second most common malignancy and the fifth leading cause of cancer-related deaths in China (https://www.iarc.who.int/faq/latest-global-cancer-data-2020-qa/).

The oncogene human epidermal growth factor receptor 2 (HER2) is localized in chromosome 17q21 and coding transmembrane tyrosine kinase receptor; its amplification plays a pivotal role in oncogenesis and development of human malignancies. HER2 has been identified to be overexpressed and/or amplified in up to 30% of breast cancers (BCs) (1) and 13% of gastroesophageal adenocarcinoma (GEA) (2). HER2-targeted therapy significantly prolongs the survival of patients with HER2-positive BC or advanced gastric cancer, which has become a basic strategy for the first-line treatment of related tumors. Recently, HER2 was also reported to be overexpressed in CRC, with some reporting expression varying in the range of 2.6%–11.2% in China (3–6). In metastatic CRC (mCRC), adding trastuzumab and pertuzumab, two monoclonal antibodies targeting HER2, to the standard chemotherapy can significantly improve response rates and survival in patients with HER2-positive tumors (7, 8). However, there is a wide variation in the HER2 positivity rate, which may be due to territorial diversity, different testing methods, and, more importantly, different scoring criteria for HER2 positivity. Previously, the data regarding HER2 expression in CRC mostly refer to HER2 scoring criteria for BC (American Society of Clinical Oncology/College of American Pathologists (ASCO/CAP) 2013) (9) and GEA (GEA criteria) (10). In 2015, Valtorta et al. (11) proposed HER2 test criteria (HERACLES diagnostic criteria) for CRC, which is now used by the National Comprehensive Cancer Network (NCCN) and the Chinese Society of Clinical Oncology (CSCO) guidelines as the diagnostic criteria for HER2 interpretation in CRC. However, there is still a lack of support from large-scale research data on whether these immunohistochemistry (IHC) interpretation criteria can accurately reflect the HER2 status in CRC in China. In addition, the correlation between HER2 and the clinicopathological parameters and prognosis in CRC remains controversial (3–6). These indicate that the positivity and role of HER2 in CRC require further exploration.

In the current study, to detect HER2 overexpression and amplification in patients with CRC (HOLIC), we carried out a large-scale retrospective study in the Chinese population. We aimed to compare the prevalence of HER2 positivity underlying the three HER2 diagnostic criteria (BC, GEA, and HERACLES diagnostic criteria). In addition, we identified HER2 expression status and its correlation with clinicopathological features as well as survival data in patients with CRC.

## Materials and Methods

### Patient Collection and Sample Preparation

A total of 4,836 consecutive cases of CRC treated by radical surgery without any preoperative therapy during 2011–2014 were retrieved from the Department of Pathology of Fudan University Shanghai Cancer Center (FUSCC). The clinicopathological characteristics and molecular pathological detection analysis results (if any) of each patient were retrieved from the hospital information systems. Among all cases, 59 patients underwent a commercial next-generation sequencing (NGS) detection in the Department of Molecular Pathology. Genomic mutations were identified using the NGS-based YuanSu450 gene panel (OrigiMed, Shanghai, China), which covers the coding exons of 450 cancer-related genes and 64 selected introns in 39 genes that are frequently rearranged in solid tumors. The genes were captured and sequenced with a mean depth of 800× using the Illumina NextSeq 500. Genomic alterations (GAs), including single-nucleotide variants (SNVs), insertion–deletion polymorphisms (Indels), and copy number variation, were identified using MuTect (v.1.17), PINDEL (v.2.04), and Control-FREEC (v9.4), respectively. The pathologic diagnosis, including the pTNM stage, was confirmed by two experienced and independent pathologists. For each sample, 3-µm sections were cut from the selected representative paraffin-embedded tumor blocks for IHC and dual-color silver-enhanced *in situ* hybridization (DSISH) analyses. The study was approved by the Ethical Committee for Clinical Research at FUSCC.

IHC staining was carried out using the anti-HER2/NEU (4B5) antibody (Ventana Medical Systems, Inc., Tucson, AZ, USA) as the primary antibody against HER2 on a Ventana Benchmark XT automatic staining system according to the optimized manufacturer’s instructions. Two pathologists independently scored HER2 staining based on both the traditional HER2 IHC scoring guidelines for BC and GEA and the HERACLES criteria for CRC (11), and any discrepancy was resolved by a consensus meeting.

To screen for deficient DNA mismatch repair (dMMR), all eligible patients had their paraffin-embedded tumor tissues tested by IHC for the expression of MLH1, MSH2, MSH6, and PMS2. dMMR was considered when there was nuclear negativity in IHC staining for MLH1, MSH2, MSH6, or PMS2, with the presence of protein expression in adjacent normal colonic mucosa and/or stromal cells.

### Dual-Color Silver-Enhanced *In Situ* Hybridization

HER2 amplification was confirmed by DSISH assays. DSISH was carried out on a Ventana Benchmark XT automatic staining system according to Chromosome 17 (CHR17) Probe (Ventana Medical Systems SA). Briefly, 20 tumor nuclei were initially counted per case. The scoring and evaluation for *in situ* hybridization were performed by counting ERBB2 and CEN17 signals from 100 nuclei per case. Non-tumor tissue (normal colon mucosa) was used as an internal negative control. Samples with an ERBB2/CEN17 ratio ≥2.0 were considered amplified (11). The DSISH was assessed by two independent pathologists separately who were blinded to the IHC scores.

### Statistical Analysis

Data were analyzed using SPSS 23.0 software. The correlations between HER2 expression and the investigated parameters were evaluated. Survival analysis was carried out using the Kaplan–Meier method, and the multivariate survival analysis was in proportional hazards regression models described by Cox. The significance tests were two-sided, and a p-value of <0.05 was considered statistically significant.

## Results

### Human Epidermal Growth Factor Receptor 2 Expression Rate Determined by Immunohistochemistry and Dual-Color Silver-Enhanced *In Situ* Hybridization

**Figure 1:**
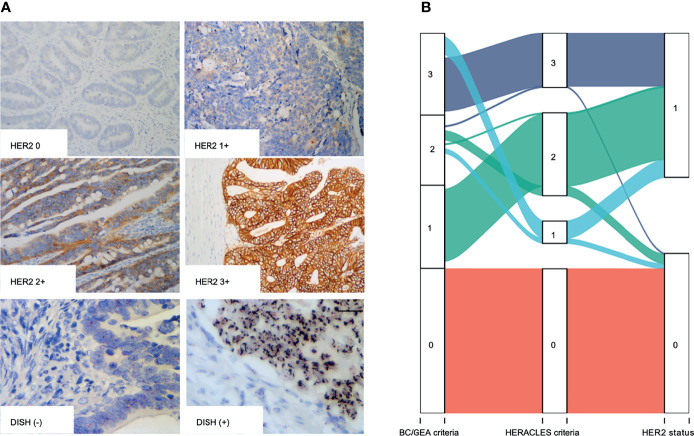
IHC and DSISH staining of HER2. **(A)** Representative IHC intensity (0, 1+, 2+, and 3+) of tumor cells (original magnification, ×200) and representative negative (DSISH−) and positive (DSISH+) amplification of HER2 gene (original magnification, ×400). **(B)** Alluvial diagram shows the distribution of each case in different diagnostic criteria. IHC, immunohistochemistry; DSISH, dual-color silver-enhanced *in situ* hybridization; HER2, human epidermal growth factor receptor 2.

The expression was studied using whole tissue sections. HER2 was observed by lateral or basolateral membrane staining in CRC cells (**Figure 1A**). According to the BC and GEA HER2 IHC diagnostic criteria, 98 (2.03%) were scored up to HER2 1+, 266 (5.50%) were scored up to but no higher than HER2 2+, and 73 cases (1.51%) were scored up to HER2 3+. Immunonegative cancer cells (HER2 0) were found in 4,399 (90.96%) cases. Thus, the HER2 expression rate of IHC (1+~3+) was 7.01% (339/4,836) in CRCs based on the BC/GEA criteria. However, there was a significant difference between the evaluation results based on the HERACLES criteria and evaluation results based on BC/GEA criteria (p < 0.001; **Figure 1B** and **Table 1**). Specifically, 47 samples scored up to but no higher than HER2 2+ according to the BC/GEA criteria was scored up to but no higher than 1+ according to the HERACLES criteria; 3 samples scored up to HER2 3+ according to the BC/GEA criteria was scored up to but not higher than 2+ according to the HERACLES criteria (**Figure 1B**). Therefore, there was 6.02% (291/4,836) of CRC scored up to HER2 2~3+ according to the HERACLES criteria, which is 0.96% less than that of BC/GEA criteria.

**Table 1:** HER2 expression in different criteria and determination methods.

Criteria		HERACLES criteria				p-Value
		0	1	2	3	
BC/GEA criteria	0	4,399	0	0	0	<0.001*
	1	0	98	0	0	
	2	0	47	219	0	
	3	0	0	3	70	
DSISH	0	/	28	149	4	<0.001*
	1	/	19	73	66	
NGS	0	/	8	28	1	<0.001*
	1	/	0	1	21	

BC, breast cancer; GEA, gastroesophageal adenocarcinoma; DSISH, dual-color silver-enhanced in situ hybridization; NGS, next-generation sequencing.

^*^All statistical tests were 2-sided; significance level: p < 0.05.

All the 339 cases that showed membranous staining of HER2 2~3+ according to BC/GEA criteria were then subjected to HER2 DSISH analysis. In addition, 50 cases displaying no/weak membranous IHC staining according to BC/GEA criteria were randomly selected for DSISH analysis, and none of these cases showed HER2 gene amplification (**Figure 1A**). As a result, 181 cases were reported as negative (not amplified), and 158 cases were reported as positive (amplified) by DSISH. Very high consistency in the results between IHC 3+ and DSISH was obtained; 67 (91.78%) of 73 HER2 IHC 3+ cases showed HER2 gene amplification. However, only 91 (34.21%) of 266 HER2 IHC 2+ cases showed HER2 gene amplification (**Figure 1B** and **Table 1**). Thus, the HER2 amplification/overexpression ratio was 3.39% (164/4,836) in our cohort. Among these HER2 gene amplification cases reported by DSISH, 19 cases were scored up to HER2 IHC 1+, and 75 cases were scored up to HER2 IHC 2+ according to the HERACLES criteria (**Table 1**).

### Association of Human Epidermal Growth Factor Receptor 2 Expression With Clinicopathological Characteristics and Survival of Patients

To determine the association of HER2 expression with clinicopathological characteristics, all 339 cases enrolled in DSISH analysis were firstly analyzed. Interestingly, in these 339 HER2 IHC 2+~3+ cases, the 158 HER2 DSISH amplified cases were more likely to be distributed in the left colon (p = 0.014), with more regional lymph nodes (p = 0.003) and vascular invasion (p = 0.013) and higher TNM stage (p = 0.029), and were mutually exclusive with dMMR (p = 0.030, **Table 2**).

**Table 2:** Correlation between HER2 expression and clinical parameters in the IHC HER2 2~3+ group (n = 339).

Variables		HER2 status^a^		p-Value
		Negative (n = 181)	Positive (n = 158)	
Age	<60	89	89	0.188
	≥60	92	69	
Gender	Male	121	98	0.354
	Female	60	60	
Tumor location	Right	50	26	0.014*
	Left	131	132	
Tumor location	Right	50	26	0.048*
	Left	39	40	
	Rectum	92	92	
Depth of infiltration (T stage)	T1	5	9	0.109
	T2	31	15	
	T3	108	95	
	T4	37	39	
Reginal lymph nodes (N stage)	N0	103	64	0.003*
	N1	78	94	
Distance metastasis (M stage)	M0	173	144	0.098
	M1	8	14	
TNM stage	Stage I	29	15	0.029*
	Stage II	69	47	
	Stage III	75	82	
	Stage IV	8	14	
Differentiation	High	3	1	0.675
	Moderate	137	122	
	Low	41	35	
Vascular invasion	Negative	140	103	0.013*
	Positive	41	55	
Perineural invasion	Negative	143	111	0.064
	Positive	38	47	
MMR status	dMMR	8	1	0.030*
	pMMR	173	157	

HER2, human epidermal growth factor receptor 2; IHC, immunohistochemistry; MMR, mismatch repair; dMMR, deficient DNA mismatch repair; pMMR, proficient mismatch repair; DSISH, dual-color silver-enhanced in situ hybridization.

a HER2 status was identified by DSISH.

^*^All statistical tests were 2-sided; significance level: p < 0.05.

For survival analysis, the follow-up information of all 164 HER2 amplification/overexpression patients and finally tumor-specific survival data in a total of 122 patients was obtained. Subsequently, 135 patients from the total 4,399 HER2-negative cases were matched using the propensity score matching (PSM) method. The correlation of HER2 expression with clinicopathological characteristics in these 257 patients was also analyzed. The result showed that regardless of the evaluation criterion used, HER2 expression was positively correlated with the female gender (all p < 0.05). However, after categorizing HER2 expression status as negative or positive, there were no statistically significant associations with any clinicopathological data (**Table 3**).

**Table 3:** Correlation between HER2 expression and clinical parameters in the matched group (n = 257).

Variables		HERACLES criteria				p-Value	BC/GEA criteria				p-Value	HER2 status^a^		p-Value
		0	1+	2+	3+		0	1+	2+	3+		Negative	Positive	
Gender	Male	83	11	35	35	0.018^*^	83	2	44	35	0.040^*^	88	76	0.518
	Female	39	8	35	11		39	2	40	12		46	47	
Age	<60	72	7	42	27	0.303	72	1	47	28	0.571	77	71	0.966
	≥60	50	12	28	19		50	3	37	19		57	52	
Tumor location	Right	20	0	13	10	0.185	20	0	13	10	0.658	21	22	0.635
	Left	102	19	57	36		102	4	71	37		113	101	
Tumor location	Right	20	0	13	10	0.264	20	0	13	10	0.526	21	22	0.723
	Left	23	7	18	9		23	2	23	9		28	29	
	Rectum	79	12	39	27		79	2	48	28		85	72	
Depth of infiltration (T stage)	T1	4	0	5	2	0.752	4	0	5	2	0.784	5	6	0.916
	T2	9	2	8	4		9	1	9	4		11	12	
	T3	79	11	35	28		79	2	43	29		82	71	
	T4	30	6	22	12		30	1	27	12		36	34	
Reginal lymph nodes (N stage)	N0	46	6	36	18	0.217	46	3	39	18	0.307	53	53	0.565
	N1	76	13	34	28		76	1	45	29		81	70	
Distance metastasis (M stage)	M0	117	17	64	40		117	3	77	41	0.120	126	112	0.363
	M1	5	2	6	6		5	1	7	6		8	11	
TNM stage	Stage I	8	1	10	4	0.213	8	1	10	4	0.197	11	12	0.682
	Stage II	37	5	24	13		37	2	27	13		40	39	
	Stage III	72	11	30	23		72	0	40	24		75	61	
	Stage IV	5	2	6	6		5	1	7	6		8	11	
Differentiation	High	2	0	0	1	0.325	2	0	0	1	0.607	2	1	0.854
	Moderate	90	16	57	31		90	3	69	32		100	94	
	Low	30	3	13	14		30	1	15	14		32	28	
Vascular invasion	Negative	82	12	53	29	0.562	82	4	61	29	0.308	93	83	0.740
	Positive	40	7	17	17		40	0	23	18		41	40	
Perineural invasion	Negative	85	16	56	31	0.223	85	4	68	31	0.109	96	92	0.569
	Positive	37	3	14	15		37	0	16	16		38	31	
MMR status	dMMR	1	0	0	0	0.774	1	0	0	0	0.774	1	0	0.337
	pMMR	121	19	70	46		121	4	84	47		133	123	

HER2, human epidermal growth factor receptor 2; BC, breast cancer; GEA, gastroesophageal adenocarcinoma; MMR, mismatch repair; dMMR, deficient DNA mismatch repair; pMMR, proficient mismatch repair; IHC, immunohistochemistry; DSISH, dual-color silver-enhanced in situ hybridization.

a HER2 status was identified by IHC and DSISH.

^*^All statistical tests were 2-sided; significance level: p < 0.05.

**Figure 2:**
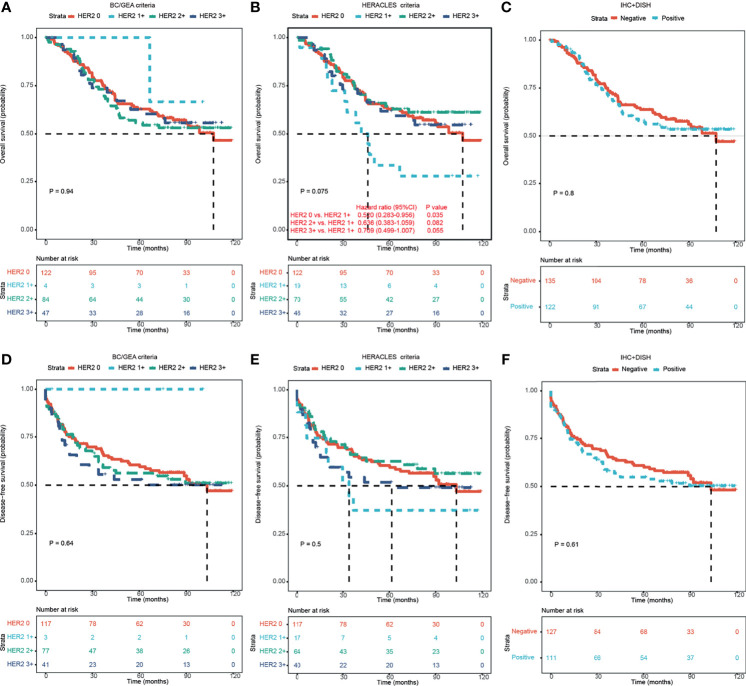
Kaplan–Meier survival analyses of HER2 expression in CRC patients according to different criteria. **(A–C)** Kaplan–Meier OS analysis of HER2 expression in CRC patients according to BC/GEA criteria **(A)**, HERACLES criteria **(B)**, and HER2 expression status **(C)**. **(D–F)** Kaplan–Meier DFS analysis of HER2 expression in CRC patients according to BC/GEA criteria **(D)**, HERACLES criteria **(E)**, and HER2 expression status **(F)**. HER2, human epidermal growth factor receptor 2; CRC, colorectal cancer; OS, overall survival; BC, breast cancer; GEA, gastroesophageal adenocarcinoma; DFS, disease-free survival.

The median follow-up time was 75.17 months (range: 0.23–119.27 months), and 113 patients (44%) died of cancer, while the remaining 144 patients (56%) were still alive at the end of the study period. A total of 123 patients (47.1%) patients relapsed; 52.1% of patients survived without a tumor. There was no significant correlation between HER2-positive and HER2-negative patients on either overall survival (OS; **Figures 2A** **–C**) or disease-free survival (DFS; **Figures 2D** **–F**).

**Figure 3:**
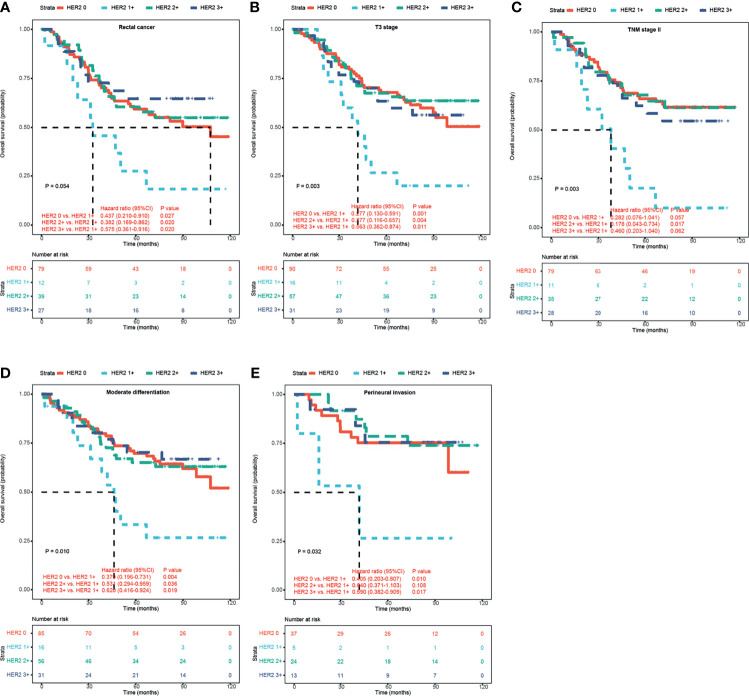
Kaplan–Meier survival analyses of HER2 expression in CRC patients according to HERACLES criteria. Kaplan–Meier OS analysis of patients with rectal cancer **(A)**, T3 stage **(B)**, TNM stage II **(C)**, moderate differentiation **(D)**, and perineural invasion **(E)** based on HER2 expression status. HER2, human epidermal growth factor receptor 2; CRC, colorectal cancer.

Interestingly, according to the HERACLES criteria, patients with HER2 1+ showed significantly shorter OS than patients with the other three HER2 expression statuses (p = 0.035 vs. HER2 0, p = 0.082 vs. HER2 2+, and p = 0.055 vs. HER2 3+; **Figure 2B**). According to the HERACLES criteria, when patients with clinicopathological characteristics were categorized, patients with HER2 1+ showed significantly shorter OS than patients with the other three HER2 expression statuses in the rectal location, T3 stage, TNM stage II, moderate differentiation, and perineural invasion groups (**Figure 3**).

### Human Epidermal Growth Factor Receptor 2 and Common Gene Status Alteration

Among these 164 cases that were determined as HER2 positive by DSISH/IHC, KRAS, NRAS, and BRAF (RAS/BRAF) mutation data determined by a commercialized NGS method were available in a total of 59 patients from the clinical molecular pathology data. Among them, 23 (38.98%) HER2-positive cases showed wild-type RAS/BRAF status. In the other 39 cases with RAS/BRAF mutations, 33 (20.63%) harbored a KRAS mutation, 3 (1.88%) harbored an NRAS mutation, and 4 (2.5%) harbored a BRAF mutation.

**Figure 4:**
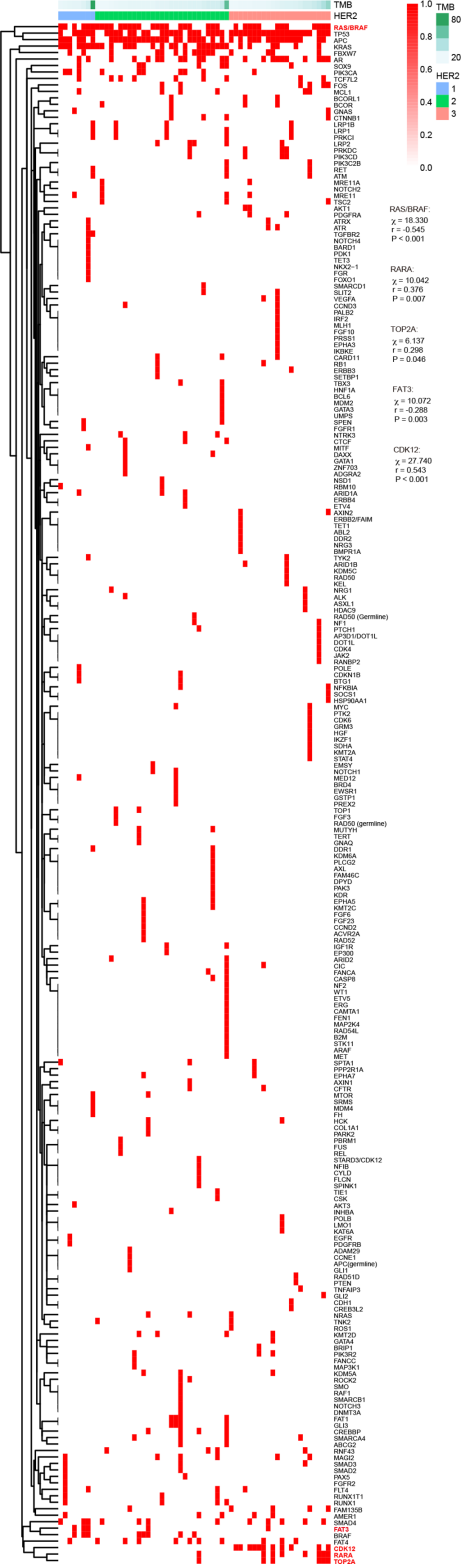
Association of HER2 expression with TMB and gene mutation according to different criteria. HER2, human epidermal growth factor receptor 2; TMB, tumor mutational burden.

In these 59 cases with second-generation sequencing data, the protein expression of HER2 according to the HERACLES criteria was as follows: 8 cases of HER2 1+, 29 cases of HER2 2+, and 22 cases of HER2 3+. Further chi-square test showed that HER2 protein expression was significantly correlated with RAS/BRAF, RARA, TOP2A, FAT3, BRAF, and CDK12 alterations (**Figure 4**). In particular, the HER2 protein expression was significantly negatively correlated with RAS/BRAF and FAT3 mutation whereas positively correlated with RARA, TOP2A, and CDK12 alterations. Among these alterations, RAS, BRAF, and FAT3 are involved in gene mutations; RARA and TOP2A are involved in gene amplification; and CDK12 are involved in both gene mutation and amplification. By classifying these patients into two groups using tumor mutational burden (TMB) >10 (12) or >28 (13) as the cutoff value, no significant correlation between TMB and HER2 protein expression was found.

The HER2 status was also determined by NGS in these 59 cases. However, it was found that for HER2 amplification, the agreement rate between DSISH and NGS was only 37.9%; only 22 HER2-positive cases also showed HER2 amplification in NGS results. By NGS, 21 cases of HER2 3+ scored by the HERACLES criteria was determined as HER2 positive, whereas all 8 cases of HER2 1+ and 28 cases of HER2 2+ scored by the HERACLES criteria were determined as HER2 negative, with a significant statistical difference (p < 0.001; **Table 1**).

## Discussion

In this study, we detected the HER2 status in CRC patients on a large scale with a total of 4,836 patients hospitalized during 2011–2014. We found that there was a significant difference between the evaluation results based on the HERACLES criteria and BC/GEA criteria. In particular, 47 samples scored as 2+ determined by BC/GEA criteria would be scored down to 1+ determined by the HERACLES criteria. In the general diagnosis process of HER2 detection, these cases would not be subsequently detected by DSISH to verify the HER2 status. However, in fact, we performed DSISH analysis in these HER2 1+ cases determined by the HERACLES criteria, and some of the cases (19/47) turned out to be HER2 positive. As we all know, DSISH is the gold standard for detecting the HER2 status. Our results suggested that relying solely on the HERACLES criteria to interpret the HER2 status might lead to missing a subset of HER2-positive CRC patients and deprive them of anti-HER2 therapy. On the other hand, for HER2 amplification, the agreement rate between DSISH and NGS was only 38.9%; only 23 positive cases showed consistent results with NGS results. It is suggested that NGS may be less sensitive to HER2-positive detection than IHC/DSISH of tissues; larger samples are needed for further validation.

The relationship between HER2 overexpression and clinicopathological features in CRC is still unclear. Meng et al. (14) found no correlation between HER2 positivity and clinicopathological parameters. Zhang et al. (6) showed that in Chinese patients with stage I to III CRC, HER2 amplification was associated with deeper intestinal wall tumor invasion and later TNM staging. In our cohort, the HER2 status has no significant association with clinicopathological features except for gender. We found that HER2 expression detected by IHC was positively correlated with the female gender, while there is no difference when we categorized the HER2 status as negative or positive based on the results of DSISH. The reasons for the inconsistent results may be attributed to about 55% of HER2 2~3+ male cases being identified as HER2 negative subsequently by DSISH, and to some extent, the percent of female cases increased in the HER2-positive group. Interestingly, when focused on those 339 patients who were scored as HER2 2~3+ according to BC/GEA criteria and then determined by DSISH, the clinicopathological feature distribution in these 339 patients showed that HER2 amplification was positively associated with tumor invasion, lymphatic metastasis, and advanced TNM staging. This suggested that once HER2 becomes expressed in CRC (which was determined by IHC), HER2 amplification is more likely to appear in patients with advanced clinical stages.

In our cohort, we obtained KRAS, NRAS, and BRAF gene statuses (determined by NGS) of 59 HER2-positive cases from their clinical data and found that 36 (38.98%) HER2-positive cases have RAS/BRAF mutation. According to the results of the Mypathway study (15), trastuzumab combined with pertuzumab treatment in RAS/RAF wild-type patients tends to obtain a higher overall response rate (ORR) efficiency, which suggests that the combined detection of HER2 and RAS/BRAF status can help in screening patients who may benefit from dual-targeted anti-HER2 therapy. It has been reported that HER2-positive and KRAS, NRAS, and BRAF mutations are mutually exclusive in CRC (16). Similarly, when determining the HER2 status according to the HERACLES criteria, the HER2 protein expression was also significantly negatively correlated with RAS/BRAF status in our cohort. This indicates that HER2-positive CRC patients are more likely to be RAS/RAF wild-type. Some studies have found that activation of HER2 signaling causes drug resistance to anti-EGFR therapy in a fraction of mCRC patients with RAS wild-type tumors (17, 18). Kras gain-of-function is one of the most frequent mutations in colon cancers, which has been shown to correlate well with poorer survival. Whether HER2 has a regulatory role in CRC needs to be further studied. Besides, the HER2 protein expression was also significantly negatively correlated with FAT3 alterations whereas positively correlated with RARA, TOP2A, and CDK12 alterations. Although the functional mechanisms behind these associations are still unclear, they provide some perspectives for the compatibility of targeted drugs.

Currently, there are conflicting studies on the relationship between HER2 positivity and the prognosis of CRC. Meng et al. (14) showed that compared with patients with HER2-negative CRC, patients with HER2-positive CRC had worse 5-year DFS and OS in the overall group of 119 patients with CRC. Liu et al. (3) also found that patients with HER2-positive CRC showed an association with shorter DFS in patients with stage II–III CRC. On the contrary, Wang et al. (4) found that patients with HER2-positive CRC showed no association with DFS or OS in the overall group of 1,058 patients with CRC. Similarly, by matching 122 HER2-positive patients with 135 HER2-negative patients using the PSM method, we found no significant correlations between HER2-positive rates and either DFS or OS regardless of the evaluation criterion used. The different research conclusions may be affected by the treatment that the patients received as well as the involved samples. In our study, firstly, we used 254 cases to ensure sufficient power to test, which is to our knowledge the largest sample size to analyze the prognostic significance in CRC. Secondly, we used the PSM to control our known confounding bias as much as possible to verify this conclusion, with a higher level of evidence. Our results might be closer to the real situation.

Interestingly, according to HERACLES diagnostic criteria, patients with HER2 1+ CRC showed the shortest OS as compared with any other group of patients. In the rectal location, T3 stage, TNM stage II, medium differentiation, and perineural invasion stratified group, the same conclusion is suggested. It is well known that HER2 can promote tumor cell proliferation, survival, and angiogenesis by activating downstream oncogenic signaling pathways (19). Therefore, it is not difficult to understand that the prognosis of HER2 1+ patients is worse than that of HER2 0 patients. In addition, consistent with the previous study (16), our study also showed that the RAS/BRAF mutation rate of patients with HER2 1+ is significantly higher than that of patients with HER2 2~3+. Although we failed to obtain the clinical therapeutic data of these patients with HER2 1~3+, in clinical practice, RAS/BRAF wild-type patients are routinely treated with EGFR monoclonal antibodies such as Erbitux. We suspect that this may be the reason why the prognosis of HER2 1+ patients is worse than that of HER2 2~3+ patients. Although our sample size of HER2 1+ patients is small, based on the clinical benefit of HER2 1+, our results suggested that special attention should be paid to patients with HER2 low-expressing CRC. More future studies are needed to break the treatment bottleneck of this subgroup of CRC patients with relatively poor biological behavior.

In recent years, a number of clinical studies have shown that dual-target anti-HER2 therapy, novel anti-HER2 tyrosine kinase inhibitor, antibody–drug conjugate (ADC) drugs, and bispecific antibodies might become a promising anti-HER2 treatment in HER2-positive mCRC (15, 20). Among these trials, DESTINY-CRC01 is the only trial that enrolled patients with HER2 low-expressing mCRC (21). The previous study has demonstrated a promising preliminary antitumor activity of T-DXd in patients with HER2 low BC (objective response rate: 37.0%) (22). However, no patients with HER2 2+ or HER2 1+ mCRC had a confirmed objective response to T-DXd.

Limitations of the study include that the treatment information of the patients is not obtained. There was a lack of efficacy data of anti-HER2 treatment. Additionally, we did not perform NGS analysis in the matching peripheral blood to verify the consistency of HER2 amplification with tissue. A recent clinical trial showed that identification of the HER2 status by circulating tumor DNA (ctDNA) showed similar accuracy to conventional tissue genotyping (23). Although this suggests that ctDNA has good application value in identifying patients who benefit from the dual-HER2 blockade and monitor treatment response, it is still necessary to conduct a large number and detailed comparative analyses on the expression profile of HER2 in tissues and ctDNA and to comprehensively judge the clinical applicability of ctDNA genotyping on HER2 amplification.

In summary, we conducted a large-scale HER2 status detection in the Chinese CRC patients and found that when scored by the HERACLES Criteria, patients with HER2 1+ CRC had the worst prognosis. Also, our study suggested that the current most commonly used HERACLES criteria might be too strict for patients with CRC. Future studies are needed to explore the most suitable criteria for screening CRC patients who could benefit from anti-HER2 therapy as much as possible.

## Data Availability Statement

The original contributions presented in the study are included in the article/Supplementary Materials, further inquiries can be directed to the corresponding author/s.

## Ethics Statement

The studies involving human participants were reviewed and approved by Ethical Committee for Clinical Research at Fudan University Shanghai Cancer Center. The patients/participants provided their written informed consent to participate in this study.

## Author Contributions

SN and XiW conceived the study, performed the literature search and bioinformatics analysis, and prepared the figures. XuW, WW, TC, MZ, and JC helped with data collection, analysis, and interpretation. MX, ZH, CJ, WW, and DH wrote and revised the manuscript. The authors read and approved the final manuscript.

## Funding

This work was supported by the National Natural Science Foundation of China (81972249, 81802367, 81802361, and 82172702), Clinical Research Project of Shanghai Shenkang Hospital Development Center (SHDC2020CR4068), Shanghai Clinical Science and Technology Innovation Project of Municipal Hospital (SHDC12020102), Fudan University Double First-class Original Research Personalized Support Project (XM03190634), the Natural Science Foundation of Shanghai (21ZR1414900), Shanghai Science and Technology Development Fund (19MC1911000), Clinical Research Project of Shanghai Municipal Health Committee (20194Y0348), Shanghai “Rising Stars of Medical Talents” Youth Development Program Youth Medical Talents—Specialist Program (SHWSRS (2020)_087) and Shanghai Anticancer Association EYAS project (SACA-CY19B10).

## Conflict of Interest

The authors declare that the research was conducted in the absence of any commercial or financial relationships that could be construed as a potential conflict of interest.

## Publisher’s Note

All claims expressed in this article are solely those of the authors and do not necessarily represent those of their affiliated organizations, or those of the publisher, the editors and the reviewers. Any product that may be evaluated in this article, or claim that may be made by its manufacturer, is not guaranteed or endorsed by the publisher.

## References

[1] Slamon DJ Clark GM Wong SG Levin WJ Ullrich A McGuire WL. Human Breast Cancer: Correlation of Relapse and Survival With Amplification of the HER-2/Neu Oncogene. Science (1987) 235(4785):177–82. doi: 10.1126/science.3798106 3798106

[2] Sheng WQ Huang D Ying JM Lu N Wu HM Liu YH. HER2 Status in Gastric Cancers: A Retrospective Analysis From Four Chinese Representative Clinical Centers and Assessment of Its Prognostic Significance. Ann Oncol: Off J Eur Soc Med Oncol (2013) 24(9):2360–4. doi: 10.1093/annonc/mdt232 23788757

[3] Liu F Ren C Jin Y Xi S He C Wang F. Assessment of Two Different HER2 Scoring Systems and Clinical Relevance for Colorectal Cancer. Virchows Arch (2020) 476(3):391–8. doi: 10.1007/s00428-019-02668-9 PMC7085476 31720832

[4] Wang XY Zheng ZX Sun Y Bai YH Shi YF Zhou LX. Significance of HER2 Protein Expression and HER2 Gene Amplification in Colorectal Adenocarcinomas. World J Gastrointest Oncol (2019) 11(4):335–47. doi: 10.4251/wjgo.v11.i4.335 PMC6475672 31040898

[5] Shan L Lv Y Bai B Huang X Zhu H. Variability in HER2 Expression Between Primary Colorectal Cancer and Corresponding Metastases. J Cancer Res Clin Oncol (2018) 144(11):2275–81. doi: 10.1007/s00432-018-2744-z PMC11813305 30203148

[6] Zhang X Wu J Wang L Zhao H Li H Duan Y. HER2 and BRAF Mutation in Colorectal Cancer Patients: A Retrospective Study in Eastern China. PeerJ (2020) 8:e8602. doi: 10.7717/peerj.8602 32095377 PMC7023828

[7] Bang YJ Van Cutsem E Feyereislova A Chung HC Shen L Sawaki A. To GATI. Trastuzumab in Combination With Chemotherapy Versus Chemotherapy Alone for Treatment of HER2-Positive Advanced Gastric or Gastro-Oesophageal Junction Cancer (ToGA): A Phase 3, Open-Label, Randomised Controlled Trial. Lancet (London England) (2010) 376(9742):687–97. doi: 10.1016/S0140-6736(10)61121-X 20728210

[8] Slamon DJ Leyland-Jones B Shak S Fuchs H Paton V Bajamonde A. Use of Chemotherapy Plus a Monoclonal Antibody Against HER2 for Metastatic Breast Cancer That Overexpresses HER2. N Engl J Med (2001) 344(11):783–92. doi: 10.1056/NEJM200103153441101 11248153

[9] Kwak Y Yun S Nam SK Seo AN Lee KS Shin E. Comparative Analysis of the EGFR, HER2, C-MYC, and MET Variations in Colorectal Cancer Determined by Three Different Measures: Gene Copy Number Gain, Amplification Status and the 2013 ASCO/CAP Guideline Criterion for HER2 Testing of Breast Cancer. J Trans Med (2017) 15(1):167. doi: 10.1186/s12967-017-1265-x PMC5540452 28764718

[10] Conradi LC Styczen H Sprenger T Wolff HA Rodel C Nietert M. Frequency of HER-2 Positivity in Rectal Cancer and Prognosis. Am J Surg Pathol (2013) 37(4):522–31. doi: 10.1097/PAS.0b013e318272ff4d 23282976

[11] Valtorta E Martino C Sartore-Bianchi A Penaullt-Llorca F Viale G Risio M. Assessment of a HER2 Scoring System for Colorectal Cancer: Results From a Validation Study. Modern Pathol: an Off J United States Can Acad Pathol Inc (2015) 28(11):1481–91. doi: 10.1038/modpathol.2015.98 26449765

[12] Chalmers ZR Connelly CF Fabrizio D Gay L Ali SM Ennis R. Analysis of 100,000 Human Cancer Genomes Reveals the Landscape of Tumor Mutational Burden. Genome Med (2017) 9(1):34. doi: 10.1186/s13073-017-0424-2 28420421 PMC5395719

[13] Chen EX Jonker DJ Loree JM Kennecke HF Berry SR Couture F. Effect of Combined Immune Checkpoint Inhibition vs Best Supportive Care Alone in Patients With Advanced Colorectal Cancer: The Canadian Cancer Trials Group CO.26 Study. JAMA Oncol (2020) 6(6):831–8. doi: 10.1001/jamaoncol.2020.0910 PMC7206536 32379280

[14] Meng X Wang R Huang Z Zhang J Feng R Xu X. Human Epidermal Growth Factor Receptor-2 Expression in Locally Advanced Rectal Cancer: Association With Response to Neoadjuvant Therapy and Prognosis. Cancer Sci (2014) 105(7):818–24. doi: 10.1111/cas.12421 PMC4317932 24730770

[15] Meric-Bernstam F Hurwitz H Raghav KPS McWilliams RR Fakih M VanderWalde A. Pertuzumab Plus Trastuzumab for HER2-Amplified Metastatic Colorectal Cancer (MyPathway): An Updated Report From a Multicentre, Open-Label, Phase 2a, Multiple Basket Study. Lancet Oncol (2019) 20(4):518–30. doi: 10.1016/S1470-2045(18)30904-5 PMC6781620 30857956

[16] Cancer Genome Atlas N. Comprehensive Molecular Characterization of Human Colon and Rectal Cancer. Nat (2012) 487(7407):330–7. doi: 10.1038/nature11252 PMC3401966 22810696

[17] Bertotti A Migliardi G Galimi F Sassi F Torti D Isella C. A Molecularly Annotated Platform of Patient-Derived Xenografts (“Xenopatients”) Identifies HER2 as an Effective Therapeutic Target in Cetuximab-Resistant Colorectal Cancer. Cancer Discov (2011) 1(6):508–23. doi: 10.1158/2159-8290.cd-11-0109 22586653

[18] Tu J Yu Y Liu W Chen S. Significance of Human Epidermal Growth Factor Receptor 2 Expression in Colorectal Cancer. Exp Ther Med (2015) 9(1):17–24. doi: 10.3892/etm.2014.2063 25452770 PMC4247305

[19] Moasser MM. The Oncogene HER2: Its Signaling and Transforming Functions and Its Role in Human Cancer Pathogenesis. Oncogene (2007) 26(45):6469–87. doi: 10.1038/sj.onc.1210477 PMC3021475 17471238

[20] Sartore-Bianchi A Trusolino L Martino C Bencardino K Lonardi S Bergamo F. Dual-Targeted Therapy With Trastuzumab and Lapatinib in Treatment-Refractory, KRAS Codon 12/13 Wild-Type, HER2-Positive Metastatic Colorectal Cancer (HERACLES): A Proof-of-Concept, Multicentre, Open-Label, Phase 2 Trial. Lancet Oncol (2016) 17(6):738–46. doi: 10.1016/S1470-2045(16)00150-9 27108243

[21] Siena S Di Bartolomeo M Raghav K Masuishi T Loupakis F Kawakami H. Trastuzumab Deruxtecan (DS-8201) in Patients With HER2-Expressing Metastatic Colorectal Cancer (DESTINY-CRC01): A Multicentre, Open-Label, Phase 2 Trial. Lancet Oncol (2021) 22(6):779–89. doi: 10.1016/S1470-2045(21)00086-3 33961795

[22] Modi S Park H Murthy RK Iwata H Tamura K Tsurutani J. Antitumor Activity and Safety of Trastuzumab Deruxtecan in Patients With HER2-Low-Expressing Advanced Breast Cancer: Results From a Phase Ib Study. J Clin Oncol: Off J Am Soc Clin Oncol (2020) 38(17):1887–96. doi: 10.1200/jco.19.02318 PMC7280051 32058843

[23] Nakamura Y Okamoto W Kato T Esaki T Kato K Komatsu Y. Circulating Tumor DNA-Guided Treatment With Pertuzumab Plus Trastuzumab for HER2-Amplified Metastatic Colorectal Cancer: A Phase 2 Trial. Nat Med (2021) 27(11):1899–903. doi: 10.1038/s41591-021-01553-w PMC8604726 34764486

